# Development of a mouse monoclonal antibody for the detection of asymmetric dimethylarginine of Translocated in LipoSarcoma/FUsed in Sarcoma and its application in analyzing methylated TLS

**DOI:** 10.1186/2045-3701-4-77

**Published:** 2014-12-10

**Authors:** Kenta Fujimoto, Riki Kurokawa

**Affiliations:** Division of Gene Structure and Function, Research Center for Genomic Medicine, Saitama Medical University, 1397-1 Yamane, Hidaka-shi, Saitama, 350-1241 Japan

**Keywords:** TLS/FUS, Arginine methylation, RNA-binding protein, Long noncoding RNA, Monoclonal antibody

## Abstract

**Background:**

RNA-binding protein Translocated in LipoSarcoma/FUsed Sarcoma (TLS/FUS) is one of causative genes for familial amyotrophic lateral sclerosis (ALS). We previously identified that TLS was associated with protein arginine methyltransferase 1 (PRMT1), and four arginine residues within TLS (R216, R218, R242 and R394) were consistently dimethylated. Protein arginine methylation is involved in various cellular events such as signal transduction, transcriptional regulation and protein-protein interactions.

**Results:**

To understand the biological role of arginine methylation of RNA-binding protein, we prepared and characterized a mouse monoclonal antibody against asymmetric dimethylarginine of TLS. By cloning and screening, one stable hybridoma cell clone (2B12) producing anti-asymmetric dimethylated TLS on R216 and R218 antibody was established. The monoclonal antibody 2B12 is specific for the asymmetrically dimethylated arginine peptide and does not react with the same peptide sequence containing unmodified and symmetrically dimethylated arginine residues by dot-blot analysis. 2B12 was also validated GST tagged TLS with PRMT1 by *in vitro* arginine methylation assays. Since methylated TLS in HeLa cells and mouse and human brain protein extracts was immunoprecipitated with 2B12, we performed RNA-binding protein immunoprecipitation assays using HeLa cell lysate and this antibody. We demonstrated that the long noncoding RNA (lncRNA) transcribed from cyclin D1 promoter binds methylated TLS.

**Conclusions:**

A monoclonal antibody that is capable of detecting the methylarginine status of TLS will facilitate the molecular and cellular analysis of transcriptional regulation by lncRNA through methylated TLS, and can be used as a favorable tool for clinical diagnosis of ALS caused by TLS dysregulation.

**Electronic supplementary material:**

The online version of this article (doi:10.1186/2045-3701-4-77) contains supplementary material, which is available to authorized users.

## Background

Translocated in LipoSarcoma/FUsed in Sarcoma (TLS/FUS) was originally identified in malignant liposarcoma as a part of the chimeric fusion protein TLS-CHOP [1]. Recently, it was reported that TLS is one of causative genes for familial amyotrophic lateral sclerosis (ALS) [2, 3]. TLS is also implicated in various cellular programs such as transcription, RNA processing and DNA repair [4]. We have demonstrated that the long noncoding RNAs (lncRNAs) transcribed from the cyclin D1 (CCND1) promoter (promoter-associated noncoding RNAs: pncRNAs) bind TLS and inhibit the histone acetyltransferase activities to repress the transcription of CCND1 gene [5]. Recent studies reveal that lncRNAs regulate the transcription of target genes [6]. The precise mechanisms of transcriptional regulation by lncRNAs, however, are still unclear.

Arginine methylation is one of posttranslational modifications, and accomplished by protein arginine methyltransferases (PRMTs). Arginine residues can be monomethylated or dimethylated, and dimethylation can be both symmetric (me2s) and asymmetric (me2a). Asymmetric dimethylarginine (aDMA) is catalyzed by the type I class of PRMTs (PRMT1, 3, 4, 6 and 8), and symmetric dimethylarginine (sDMA) is catalyzed by the type II class (PRMT5 and 7). In regarding to histone arginine modification, H4R3me2a and H4R3me2s are basically linked to transcriptional activation and repression, respectively [7, 8]. We have shown that TLS is associated with PRMT1, and four arginine residues within TLS (R216, R218, R242 and R394) are constitutively dimethylated [9]. However, the functional role of arginine methylation of RNA-binding proteins still needs to be studied. RNA-binding proteins often contain glysine-arginine-rich motifs and are considered substrates for PRMTs. In fact, FMRP, EWS, which are also related with diseases, are dimethylated [10, 11]. Therefore, it is believed that methylation of RNA-binding proteins could influence RNA-protein and/or protein-protein interactions.

ALS is a fatal neurodegenerative disease caused by degeneration of motor neurons. Identification of several mutations in the TLS gene from ALS patients suggested that disruption of RNA metabolism might be one of key events in ALS pathogenesis. Interestingly, natural arginine mutation (R216C), one of methylated arginine we identified, of TLS from ALS patients was reported [12]. Moreover, it was an interesting report that the RNA-binding ability of TLS is essential for the neurodegenerative phenotype *in vivo* of mutant TLS although it was unclear whether direct contact with RNA or through interactions with other RNA-binding proteins [13]. Taken together, these findings suggest that arginine methylation of TLS might play an important role in the lncRNA-dependent transcriptional regulation and the disruption of RNA binding could be implicated in the pathogenesis of ALS.

In this study, we attempt to establish hybridoma cell lines that can stably produce anti-methylated TLS monoclonal antibodies. Here we show one monoclonal antibody (2B12) can specifically recognize arginine-methylation of TLS. Our generated antibody could detect selectively the asymmetrically dimethylated TLS by western blotting. Moreover, 2B12 was suitable for RNA-binding protein immunoprecipitation (RIP) assays to show the interplay between lncRNA and methylated TLS.

## Results

### Generation of asymmetric dimethylarginine-specific antibody and antibody specificity

**Figure 1:**
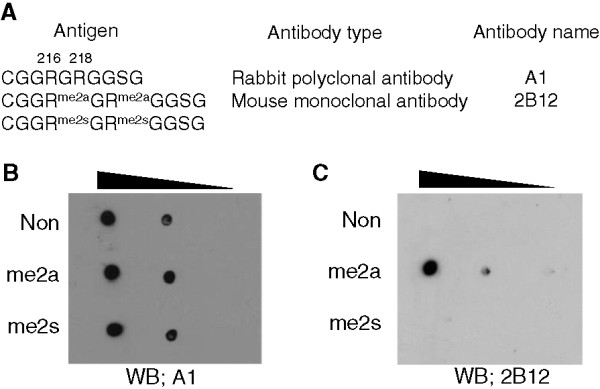
**The monoclonal antibody specificity tested by dot-blot analysis. (A)** Summary of peptide sequences. Three TLS peptides containing either no modification (Non) or R216/R218me2a (me2a) or R216/R218me2s (me2s) were synthesized. TLS peptide containing no modification was used for producing polyclonal antibody in rabbit, and TLS peptides containing R216/R218me2a or R216/R218me2s were used for the immunization of mice and hydridoma development. **(B** **and** **C)** Antibody specificity was tested by dot-blot analysis. Diluted peptides (B; 0.2, 1, 5 ng, C; 20, 100, 500 ng) were blotted onto the nitrocellulose membrane and the dot-blotted membranes were incubated with a rabbit polyclonal antibody A1 **(B)** or a mouse monoclonal antibody 2B12 **(C)**. Note that A1 reacted equally with TLS peptides either no modification or symmetrical or asymmetrical dimethylation, and 2B12 recognized only TLS peptide containing asymmetrical dimethylated arginines.

We have recently demonstrated that PRMT1 asymmetrically methylates TLS/FUS on arginine (R) residues [9]. Using mass spectrometry, we identified which residues of TLS are methylated *in vivo*[9]. To investigate the biological role of methylated TLS, we attempted to develop mouse monoclonal antibodies that specifically recognized TLS symmetrically or asymmetrically dimethylated on R216 and R218. We prepared TLS peptides that were contained unmodified, symmetrically modified (me2s), or asymmetrically modified arginines (me2a) at R216 and R218 (Figure 1A). Unmodified peptide was used for producing polyclonal antibody in rabbits, and the antiserum was obtained (hereafter referred as A1). Modified peptides were used for immunization of mice, and hybridoma clones were screened by enzyme-linked immunosorbent assay (ELISA). We obtained a few positive clones. The purified antibody (hereafter referred as 2B12) was selected for further analysis. To access antibody specificity, we tested 2B12 using synthetic peptides by dot-blot analysis. As shown in Figure 1B, A1 reacts with all of synthesized peptides equally. In contrast, the monoclonal antibody 2B12 specifically recognizes the asymmetrically methylated peptide and does not react with the same peptide sequence containing unmodified and symmetrically dimethylated arginine residues by dot-blot analysis (Figure 1C), confirming the specificity of 2B12 for asymmetric arginine methylation of TLS. Unfortunately we were not able to obtain a monoclonal antibody for detecting R216/R218me2s in this study.

### *In vitro*methylation of TLS

**Figure 2:**
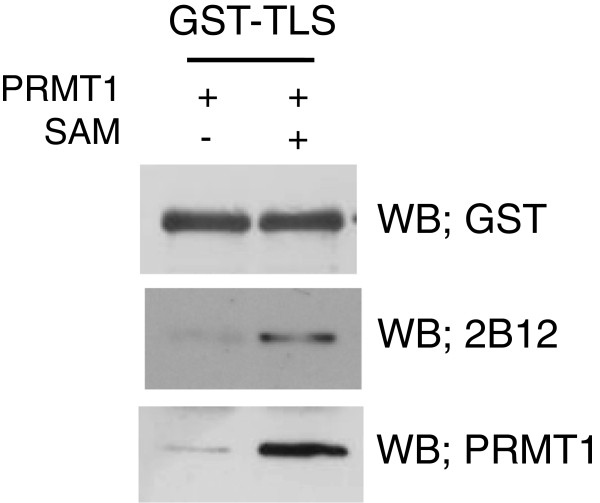
***In vitro*** **methylation of the recombinant GST-TLS.** GST-TLS was *in vitro* methylated using PRMT1 in the presence or absence of SAM (20 μM). Reaction products were analyzed by SDS-PAGE followed by western blotting with the indicated antibodies: anti-GST (top), 2B12 (middle), and anti-PRMT1 (bottom). Note that 2B12 specifically reacts with TLS methylated by PRMT1 only in the presence of SAM, and methylated TLS strongly associates with PRMT1.

To validate whether 2B12 can detect methylated TLS, we performed *in vitro* methylation assays by incubating GST tagged TLS (GST-TLS) with protein arginine methyltransferase 1 (PRMT1) as we reported previously [9]. Western blotting using 2B12 was performed, and the signal was detected in GST-TLS methylated by PRMT1 in the presence of S-adenosyl methionine (SAM) (Figure 2). No signal was observed in the absence of methylation (*i.e.* without SAM) (Figure 2). Interestingly, the interaction between TLS and PRMT1 was enhanced by the methylation of TLS (Figure 2). These results suggest that 2B12 specifically reacts with TLS methylated by PRMT1 (*i.e.* asymmetrical dimethylation), and methylation of TLS may effect protein-protein interactions.

### TLS is arginine methylated in HeLa cells

**Figure 3:**
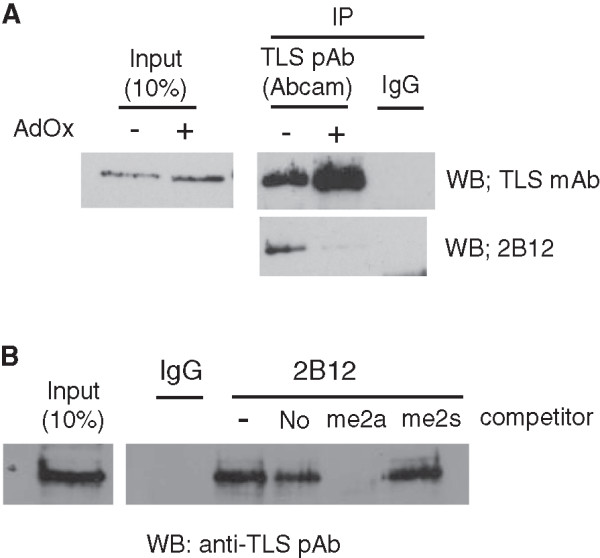
**Detection of** ***in vivo*** **methylation of TLS. (A)** Endogenous TLS is methylated in HeLa cells. Cell extracts from HeLa treated or not with 3 μM of the general methylation inhibitor AdOx for 24 h were used for immunoprecipitations. The extracts were immunoprecipitated with rabbit normal IgG or rabbit polyclonal anti-TLS antibody. The immunoprecipitated TLS were analyzed by western blotting with 2B12 or mouse monoclonal anti-TLS antibody. The input lane shows 10% of the protein used in each immunoprecipitation. Note that TLS methylation was inhibited by AdOx, and 2B12 specifically recognized methylated TLS. **(B)** Immunoprecipitation of endogenous methylated TLS from HeLa cell extracts was performed with 2B12 in the presence or absence of competing peptides used for immunization. Bound methylated TLS was eluted with SDS sample buffer resolved by SDS-PAGE, and analyzed by western blotting with rabbit polyclonal anti-TLS antibody.

To examine whether 2B12 can detect methylated TLS *in vivo*, we carried out immunoprecipitation (IP) experiments on HeLa cells. We should note that TLS was not immunoprecipitated with a rabbit polyclonal antibody A1 (data not shown). Thus, we used a rabbit polyclonal anti-TLS antibody commercially available. To verify the specificity of 2B12, HeLa cells were treated with a methyltransferase inhibitor adenosine-2’,3’-dialdehyde (AdOx) (3 μM) for 24 hours. Reduced recognition of TLS by 2B12 was observed for the AdOx-treated cell extracts, indicating that the treatment significantly reduced TLS methylation and 2B12 specifically recognized methylated TLS (Figure 3A). Somehow unmethylated TLS was immunoprecipitated with TLS polyclonal antibody efficiently although the expression levels of TLS were almost same between control and AdOx-treated cells (Figure 3A). We also assessed if 2B12 could immunoprecipitate methylated TLS *in vivo*. To test cross reactivity of 2B12, peptide inhibition assays were done. Cell extracts were immunoprecipitated with 2B12 in the presence of competing peptides used for immunization as shown in Figure 1A, and the presence of TLS was revealed using an anti-TLS polyclonal antibody. The immunoprecipitation of 2B12 was clearly inhibited by the excess of R216/R218me2a peptide in a dose-dependent manner, not by other peptides (Figure 3B and Additional file 1), indicating that a monoclonal antibody 2B12 specifically immunoprecipitated asymmetrically dimethylated TLS. These results suggest that 2B12 can be valuable to identify and investigate methylated TLS *in vivo*.

### Assessment of antibody suitability for immunoprecipitation and RIP assays

**Figure 4:**
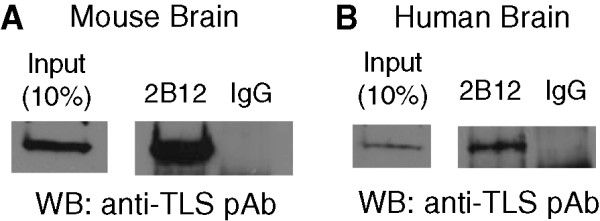
**2B12 is suitable for immunoprecipitation analysis.** 2B12 was used to immunoprecipitate methylated TLS in the total cell lysate from mouse brain **(A)** and human brain **(B)**. Bound methylated TLS was eluted with SDS sample buffer resolved by SDS-PAGE, and analyzed by western blotting with rabbit polyclonal anti-TLS antibody. The input lane shows 10% of the protein used in each immunoprecipitation.

**Figure 5:**
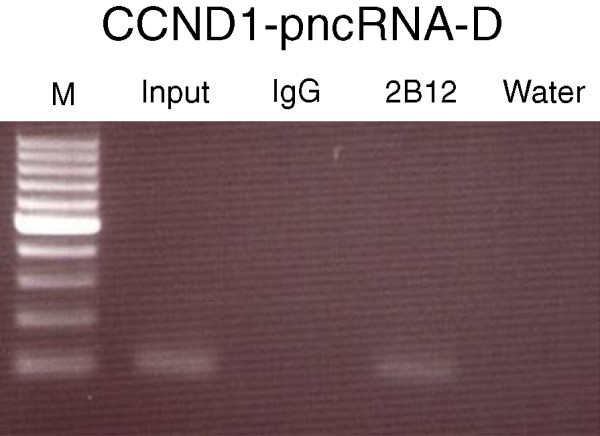
**Methylated TLS binds CCND1-pncRNA-D.** RIP lysate from HeLa cells were immunoprecipitated using either 2B12 or a normal mouse IgG as a negative control. RNA associated with methylated TLS was purified, and validated by RT-PCR using the specific primers for CCND1-pncRNA-D. The PCR prodcuts were ran on an agarose gel to detect the presence of CCND1-pncRNA-D. The “input” omits the immunoprecipitation step, “IgG” used an IgG antibody for the immunoprecipitation, “2B12” used a 2B12 antibody to pull down methylated TLS, and “water” lane served as a negative control for the PCR reaction. The expected size of PCR product for CCND1-pncRNA-D could be detected in 2B12 RIP. PCR product was observed in the 10% input and not in the normal mouse IgG RIP.

The antibody for detecting methylated TLS may be a valuable tool for analyzing the ALS pathogenesis caused by TLS dysregulation using IP and the function of TLS methylation *in vivo* using RNA-binding protein immunoprecipitation (RIP) assays. We have shown that TLS binds the lncRNAs transcribed from CCND1 promoter (CCND1 pncRNAs) [5]. The importance of arginine methylation of TLS for RNA-protein interactions needs to be studied. RIP assay is a powerful technique for studying RNA-binding proteins and their RNA partners. We demonstrated the specificity of 2B12 in Figures 1, 2 and 3. Thus, we carried out IP assays using mouse and human brain samples. 2B12 was able to specifically precipitate methylated TLS from mouse and human brain extracts (Figure 4). We further examined RIP assays using 2B12 for detecting the interplay between methylated TLS and lncRNA. RIP was conducted using HeLa cell lysate and either 2B12 or normal mouse IgG. Purified RNA was then analyzed by RT-PCR using the specific primers for the D region of CCND1 pncRNA (CCND1-pncRNA-D). As shown in Figure 5, PCR product was observed in the input and not in the normal mouse IgG RIP. CCND1-pncRNA-D could be detected in 2B12 RIP by RT-PCR, suggesting that CCND1-pncRNA-D binds methylated TLS *in vivo*.

## Discussion/conclusions

We previously demonstrated that CCND1 pncRNAs bind to TLS and inhibit the histone acetyltransferase activities to repress the transcription of CCND1 gene [5]. We also identified that four arginine residues within TLS (R216, R218, R242 and R394) were consistently dimethylated by a mass spectrometry [9]. These results suggest that arginine methylation of TLS could have an important role for the transcriptional regulation by lncRNA.

In this study, we attempted to establish hybridoma cell lines that can stably produce anti-methylated TLS monoclonal antibodies by hybridoma technique. By cloning and screening, one mouse monoclonal antibody specific to dimethylated TLS on R216 and R218 was obtained and the hybridoma cell line was named as 2B12. The characteristic of 2B12 was confirmed by dot-blot and western blot analyses (Figures 1 and 2). Methylated TLS is more associated with PRMT1 by *in vitro* methylation assays (Figure 2), suggesting that arginine-methylation of TLS might affect protein-protein interactions. Recently, many proteins have been reported to contain both sDMA and aDMA [14, 15]. It will be possible that TLS is also modified by the symmetric and asymmetric methylations on the same arginine residues. Since we did not obtain monoclonal antibodies against symmetrically dimethylated TLS, further studies will be required to solve this point.

TLS was originally identified as a fusion protein TLS-CHOP in myxoid liposarcoma [1]. More recently, TLS attracts attention because it was found to be a causative gene for the familial ALS [2, 3]. More than a dozen mutations were reported in amino acids sequence of TLS [16, 17]. It is interesting to note that R216, one of dimethylated arginine we identified, is the site of a naturally occurring mutation associated with ALS [12]. Thus, the posttranslational modification of TLS might be implicated in the pathogenesis of ALS. Since 2B12 was suitable to precipitate methylated TLS in mouse and human brain samples (Figure 4), 2B12 can be a favorable tool for clinical diagnosis of ALS and will gain insight into the pathogenesis of ALS caused by arginine mutations of TLS.

We also verified whether our antibody could be used for RIP assays. CCND1-pncRNA-D binds methylated TLS *in vivo* by RIP using 2B12 (Figure 5), suggesting that arginine-methylation of TLS can affect RNA-protein interactions. The antibody for detecting asymmetrical arginine-specific methylation of TLS can be a valuable tool for analyzing the function of TLS methylation *in vivo*. Further study using 2B12 will uncover the mechanism of transcriptional regulation by lncRNA via RNA-binding protein TLS.

## Methods

### Antibodies and reagents

Rabbit anti-TLS/FUS antibody (ab70381) was purchased from Abcam. Mouse monoclonal anti-TLS antibody (611384) was purchased from BD Biosciences. Rabbit anti-GST antibody (Z5; sc-459) was purchased from Santa Cruz Biotechnology. Rabbit anti-PRMT1 antibody (A33) was purchased from Cell Signaling technology. Adenosine dialdehyde (Adox, Sigma) was dissolved in DMSO. Total protein lysate from mouse brain (8–10 weeks) and human brain (66 years old) were obtained from BioChain Institute Inc (Newark, CA, USA).

### Peptide synthesis and antibody preparation

Unmethylated and methylated forms of peptides derived from TLS/FUS were obtained from Scrum Inc, (Tokyo, Japan). The sequences of the peptides were identical except for the presence of symmetric or asymmetric dimethylated arginine in peptide (See Figure 1A).

Rabbit polyclonal antibody against TLS peptide containing no modification (named as A1) was produced in Scrum Inc. The mouse monoclonal antibodies against TLS peptides containing either R216/R218me2s or R216/R218me2a were produced in ITM Co. Ltd. (Nagano, Japan). After the immunization and hydridoma development, cells were screened by enzyme-linked immunosorbent assay. One specific antibody against R216/R218me2a (hybridoma clone; 2B12) was obtained and characterized.

### In vitro methylation assay

*In vitro* methylation reactions were performed as described previously [9]. Briefly, GST tagged TLS were incubated with bacterially expressed Strep-tagged PRMT1 lysate in the presence or absence of SAM (Sigma) for 1 h at 30°C. Methylation reactions were quenched by the addition of SDS sample buffer, heated at 100°C for 2 min, and separated on SDS-PAGE followed by western blotting analysis.

### Dot-blot and western blot analyses

For the dot-blot analysis, one μl of diluted peptide in sterile water was blotted onto the nitrocellulose membrane (Bio-Rad) and dried. The membrane was then blocked with freshly prepared PBS containing 5% non-fat milk for 1 h at room temperature with constant agitation. The membrane was incubated with the primary antibodies diluted in 1% freshly prepared PBS-milk solution for 1 h at room temperature. After incubating the membrane with the secondary antibody (anti-mouse HRP-conjugated IgG, Dako or anti-rabbit HRP-conjugated IgG, Cell Signaling technology). Chemiluminescent detection was performed using SuperSignal West Pico substrate (Thermo Scientific).

For western blotting analysis, samples were separated by SDS-PAGE and the proteins were transferred to a nitrocellulose membrane. The membrane was then blocked similar to that used in the dot-blot analysis as mentioned above.

### Cell culture

HeLa cells were maintained at 37°C in Dulbecco’s modified Eagle’s medium (DMEM, Nacalai tesque, Tokyo, Japan) supplemented with 10% fetal bovine serum (Nichirei Biosciences Inc). HeLa cells were treated with AdOx (Sigma) for 24 hours. Cells were lysed in RIPA buffer, and cell lysates were used for immunoprecipitation experiments.

### Immunoprecipitation

Cell extracts from HeLa, mouse brain and human brain were incubated with appropriate antibodies as indicated. Antibodies against methylated TLS or normal IgG were incubated with Protein G magnetic Dynabeads (Life technologies) for 10 min at RT with gentle rotation. The cell extract was added to the mix and incubated for 10 min at RT with gentle rotation. Beads were collected and washed three times with WCE buffer, eluted by adding SDS-sample buffer. For peptide competition assays, cell extracts were incubated in the presence or absence of competing peptide with magnetic Dynabeads Protein G. The eluted samples were analyzed by SDS-PAGE and western blotting.

### RNA-binding protein immunoprecipitation assay

To determine whether methylated TLS interacts with lncRNA, 2B12 was used to pull down methylated TLS, and then bound RNA was purified and detected the expression of lncRNA from CCND1 promoter by RT-PCR using specific primers as published [5]. Magna RIP™ RNA-binding protein Immunoprecipitation kit (Millipore) was used for RIP procedures according to the manufacture’s protocol. The precipitated RNA was subject to cDNA synthesis. The presence of CCND1-pncRNA-D in the cDNA samples was detected using PCR primers previously used [5].

## Electronic supplementary material

Additional file 1: **Immunoprecipitation of endogenous methylated TLS from HeLa cell extracts was performed with 2B12 in the presence or absence of competing peptides (No; 100 ng, me2a; 25, 50, 100 ng, me2s; 100 ng). Bound methylated TLS was eluted with SDS sample buffer resolved by SDS-PAGE, and analyzed by western blotting with rabbit polyclonal anti-TLS antibody.** Note that the immunoprecipitation of 2B12 was inhibited by the excess of R216/R218me2a peptide in a dose-dependent manner, not by other peptides. (PPT 72 KB)

## Abbreviations

TLS: Translocated in LipoSarcoma
FUS: FUsed Sarcoma
CCND1: Cyclin D1
lncRNA: Long noncoding RNA
pncRNA: Promoter-associated ncRNA
RIP: RNA-binding protein immunoprecipitation
PRMT: Protein arginine methyltransferase
ALS: Amyotrophic lateral sclerosis
AdOx: Adenosine-2’,3’-dialdehyde
SAM: S-adenosyl methionine.
