# Supplementary figures and images for: Development of a mouse monoclonal antibody for the detection of asymmetric dimethylarginine of Translocated in LipoSarcoma/FUsed in Sarcoma and its application in analyzing methylated TLS
Source: Cell Biosci. 2014 Dec 10;4:77. doi: 10.1186/2045-3701-4-77 (PMC4373027; doi:10.1186/2045-3701-4-77)

## Slide 1
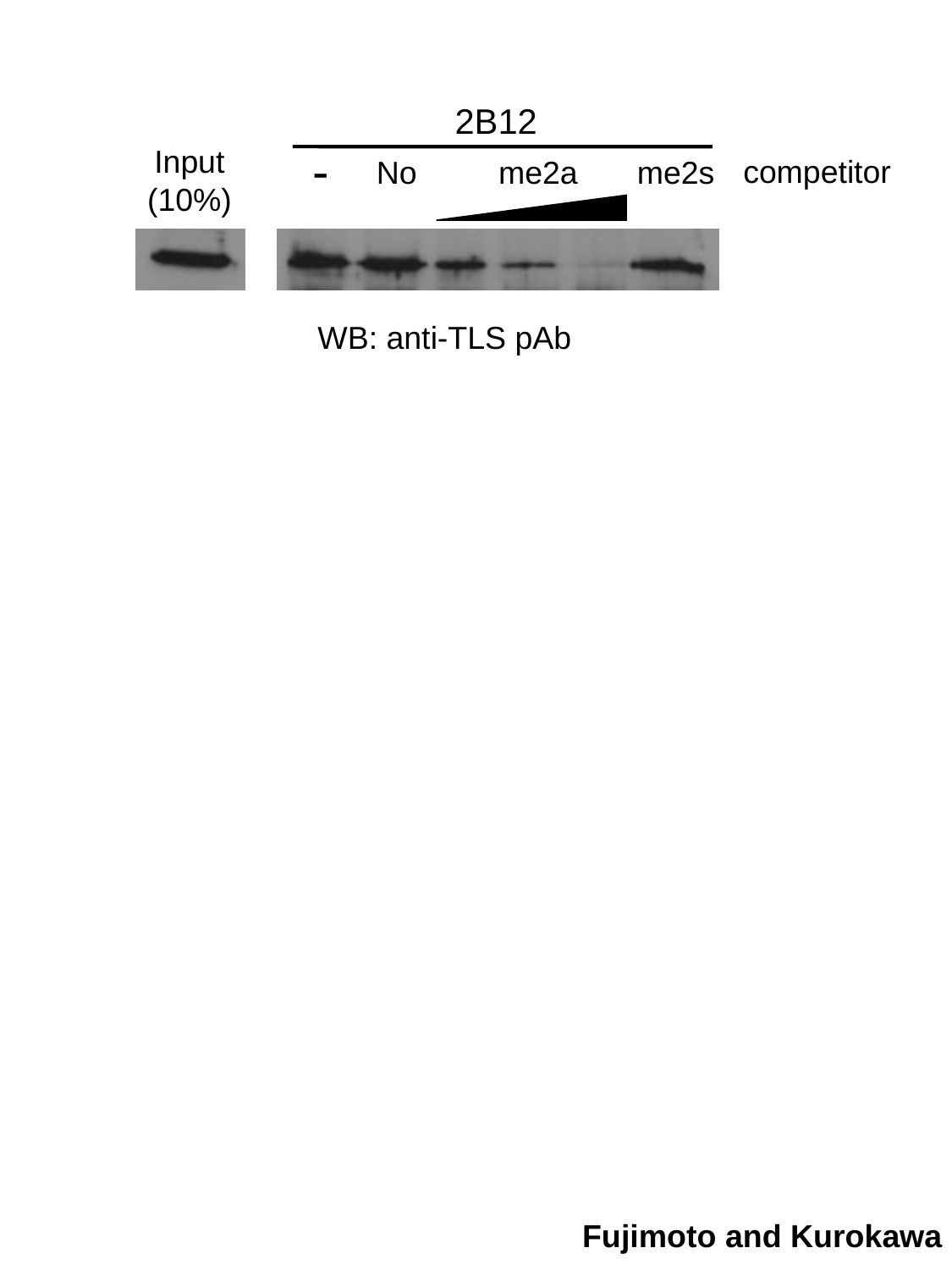

2B12
-
Input
(10%)
competitor
No
me2a
me2s
WB: anti-TLS pAb
Fujimoto and Kurokawa

Supplement: Supplementary file 1 — Additional file 1: Immunoprecipitation of endogenous methylated TLS from HeLa cell extracts was performed with 2B12 in the presence or absence of competing peptides (No; 100 ng, me2a; 25, 50, 100 ng, me2s; 100 ng). Bound methylated TLS was eluted with SDS sample buffer resolved by SDS-PAGE, and analyzed by western blotting with rabbit polyclonal anti-TLS antibody. Note that the immunoprecipitation of 2B12 was inhibited by the excess of R216/R218me2a peptide in a dose-dependent manner, not by other peptides. (PPT 72 KB) [file 13578_2014_201_MOESM1_ESM.ppt]
